# Supplementary material for: Synaptopodin KO rat for assessing the dendritic spine apparatus and axonal cisternal organelle in synaptic plasticity, development, and behavior
Source: PLoS One. 2026 Sep 3;21(9):e0355625. doi: 10.1371/journal.pone.0355625 (PMC13541181; doi:10.1371/journal.pone.0355625)
Supplement: Supplementary file 1 [file pone.0355625.s001.pdf]

S1 Figure. Original gel image used in Figure 1B.

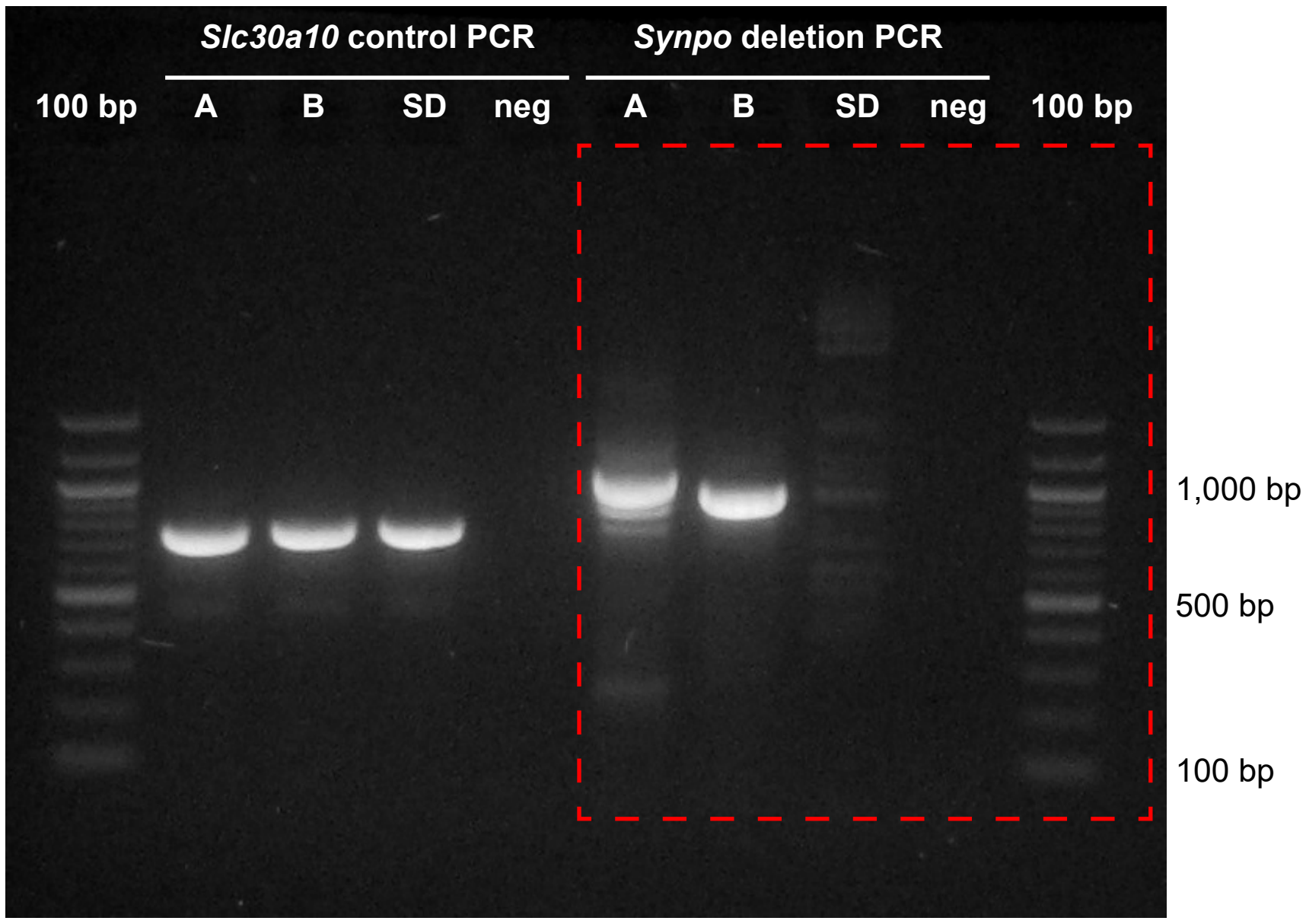

Left:
PCR products for *Slc30a10* from two founder lines (A and B), wild-type Sprague-Dawley rat (SD), and negative control (no DNA; neg), showing that DNA in the sample is intact.
The founder lines: A = LE-Synpo^{em1Kmh} and B = LE-Synpo^{em2Kmh}

Right:
PCR products for *Synpo* from two founder lines show fragments with expected sizes (A, 966 bp; B, 891 bp). For SD, PCR band is absent because the conditions do not support amplification of the longer wild-type sequence. neg = no DNA negative control.
Area indicated by the dotted red box was used in Figure 1B.
