# Supplementary material for: Synaptopodin KO rat for assessing the dendritic spine apparatus and axonal cisternal organelle in synaptic plasticity, development, and behavior
Source: PLoS One. 2026 Sep 3;21(9):e0355625. doi: 10.1371/journal.pone.0355625 (PMC13541181; doi:10.1371/journal.pone.0355625)
Supplement: Supplementary file 2 [file pone.0355625.s002.docx]

**S1 Document. Information on CRISPR Reagents and Genotyping.**

**Index**

1. CRISPR Reagents	Page 1
2. Genotyping Information
   a. LE-*Synpo^em1Kmh^*	Page 2
   b. LE-*Synpo^em2Kmh^*	Page 4

**CRISPR Reagents**

**5’ guide RNA sequence**

AAAGGTAAACACGTCAGACG

**3’ guide RNA sequence**

GTCAAGGGGCTACTCCAGTA

**Genotyping Information**

**LE-*Synpo^em1Kmh^* (also known as “synaptopodin knockout rat allele A”; RRID:RRRC_00964; RGD 155782907)**

***Wildtype***

**5’ region sequence** = 939 bp

TGGAGTGACTCAGCCTTTGCCACCTGGAGCTGTACGGCCTTGGGTCTGTCAGGAATCTCTGAACTTCTTCACACAGCTGCCCAGTGGCTGCCTCTCAGTACACTGGGGAGAACAAGATGAATGAACACAGGGAGCGTTGACATTAGCCCAGGGTATCACTTGGCTTATGTTAGCATGTACAAATTTTTAAATAATTATCTTAACAAAAGTGAGAGGAAAAAAATGGGGAAGGGTGTAGGAGAAACTGGATCGTGGTCTCTTGATTTGATGTGTTATAATTAGGGTTGGGGACACGGCTACTTTTTCATGTTTTTATTTTTTAGAACTAAAACGTGTTAACCATTTTATTTTTTGGAGACATTAGTCCTGGTTGTCCTGGAGCATGTTATGTAGACCGGGCTGGCCTAGAACTTGCAAAGATCCTCCTGCCTCTGCTTTCCCAAGTGCTGGGATTATAGGCATGTATCACCATGCCTGCATATTAATATGTTTTCCGAAAAAGAAGTCTTAGGGGCAGGAGAGATGGCTCAACAGGTAAAGGTACTTGCTGTGCAAGCCTGGTGTCTGAGTTTGATTCCCAGGACCCGTGTAAAGGTAGAACGAGACAAAATTATCCTCTGACCTCCACATGTGTAAACATGGTACATGCCTCCCCACATCAATAATGATAAATTTGAAAGGCCTTAAAAAACAACTTAGTGGCCCAGTTCTAAAATGATATCAAGCAAATGACCAAACTTTGAAAGTATAAAGAGAGAAATGCGTAAGGTGTCCTGGGCTTTGCACACTGGGGAATCTTTAGGGAATCTATTCTTGGAATTGAGGTCAGAAAGGTAAACACGTCAGACGAGGCCTTCGGAGCTGTGCTTGAGGTGAGGAGATGGATGGTCAGTCAGGAGATAAGCAAACTCGTTGATTTCGGACAGTGGTGAATGAGTG

Magenta = PCR primer targets

- 5’ Forward primer = TGGAGTGACTCAGCCTTTGC
- 5’ Reverse primer = CACTCATTCACCACTGTCCGA

Cyan = guide RNA target

Green = 5’ breakpoint

**3’ region sequence** = 483 bp

TCCCAGACCCATGCTTTTCTGGGATCATTGATGGCTAGTTTTAGGTTTTCAAAACCGGGAAGAGACGAGATGGCAGTCTAACACGGCTACCCTAGTCTAGGGTGTATTACCTATTTGGAAAGGGGTCCATGTTGCAGAATATGCAAGTCATGCCCAGGATGCGCGGAGGAGACTCCGGTATTGGGTAGAGTTGACTCCTGACCTCTGAGGATTCTTGAAACTCACTCCATAGTATTAGAAAGCAAGGGAGGGTGAAGGGGGAGGTTCACAGGGCTTGAATTCAGAGACAAACATGGGTGGGCAGTCAAGGGGCTACTCCAGTATGGGGCTGCCATAGTGTCTATATTATGCGCAGAAGAATGGGTATTGTTATATTCATTGTCTTCACATCTCTCTCTGGCCTGGCTCATACCTGTATCAGCCTTTGAGGCATCTGTCCCCTTAACCTCAGTGACTCTGATACCAGTCCCCAAGAGTGAGTCG

Magenta = PCR primer targets

- 3’ Forward primer = TCCCAGACCCATGCTTTTCT
- 3’ Reverse primer = CGACTCACTCTTGGGGACTG

Cyan = guide RNA target

Yellow = 3’ breakpoint

***Knockout***

**Deletion allele sequence** = 966 bp

TGGAGTGACTCAGCCTTTGCCACCTGGAGCTGTACGGCCTTGGGTCTGTCAGGAATCTCTGAACTTCTTCACACAGCTGCCCAGTGGCTGCCTCTCAGTACACTGGGGAGAACAAGATGAATGAACACAGGGAGCGTTGACATTAGCCCAGGGTATCACTTGGCTTATGTTAGCATGTACAAATTTTTAAATAATTATCTTAACAAAAGTGAGAGGAAAAAAATGGGGAAGGGTGTAGGAGAAACTGGATCGTGGTCTCTTGATTTGATGTGTTATAATTAGGGTTGGGGACACGGCTACTTTTTCATGTTTTTATTTTTTAGAACTAAAACGTGTTAACCATTTTATTTTTTGGAGACATTAGTCCTGGTTGTCCTGGAGCATGTTATGTAGACCGGGCTGGCCTAGAACTTGCAAAGATCCTCCTGCCTCTGCTTTCCCAAGTGCTGGGATTATAGGCATGTATCACCATGCCTGCATATTAATATGTTTTCCGAAAAAGAAGTCTTAGGGGCAGGAGAGATGGCTCAACAGGTAAAGGTACTTGCTGTGCAAGCCTGGTGTCTGAGTTTGATTCCCAGGACCCGTGTAAAGGTAGAACGAGACAAAATTATCCTCTGACCTCCACATGTGTAAACATGGTACATGCCTCCCCACATCAATAATGATAAATTTGAAAGGCCTTAAAAAACAACTTAGTGGCCCAGTTCTAAAATGATATCAAGCAAATGACCAAACTTTGAAAGTATAAAGAGAGAAATGCGTAAGGTGTCCTGGGCTTTGCACACTGGGGAATCTTTAGGGAATGGGGCTGCCATAGTGTCTATATTATGCGCAGAAGAATGGGTATTGTTATATTCATTGTCTTCACATCTCTCTCTGGCCTGGCTCATACCTGTATCAGCCTTTGAGGCATCTGTCCCCTTAACCTCAGTGACTCTGATACCAGTCCCCAAGAGTGAGTCG

Magenta = PCR primer targets

- PCR product generated using 5’ Forward and 3’ Reverse primers
- 5’ Forward primer = TGGAGTGACTCAGCCTTTGC
- 3’ Reverse primer = CGACTCACTCTTGGGGACTG

Green and Gray = 5’-3’ fusion

**Genotyping Information**

**LE-*Synpo^em2Kmh^* (also known as “synaptopodin knockout rat allele B”; RRID: RRRC_01025; RGD 616335891)**

***Wildtype***

**5’ region sequence** = 939 bp

TGGAGTGACTCAGCCTTTGCCACCTGGAGCTGTACGGCCTTGGGTCTGTCAGGAATCTCTGAACTTCTTCACACAGCTGCCCAGTGGCTGCCTCTCAGTACACTGGGGAGAACAAGATGAATGAACACAGGGAGCGTTGACATTAGCCCAGGGTATCACTTGGCTTATGTTAGCATGTACAAATTTTTAAATAATTATCTTAACAAAAGTGAGAGGAAAAAAATGGGGAAGGGTGTAGGAGAAACTGGATCGTGGTCTCTTGATTTGATGTGTTATAATTAGGGTTGGGGACACGGCTACTTTTTCATGTTTTTATTTTTTAGAACTAAAACGTGTTAACCATTTTATTTTTTGGAGACATTAGTCCTGGTTGTCCTGGAGCATGTTATGTAGACCGGGCTGGCCTAGAACTTGCAAAGATCCTCCTGCCTCTGCTTTCCCAAGTGCTGGGATTATAGGCATGTATCACCATGCCTGCATATTAATATGTTTTCCGAAAAAGAAGTCTTAGGGGCAGGAGAGATGGCTCAACAGGTAAAGGTACTTGCTGTGCAAGCCTGGTGTCTGAGTTTGATTCCCAGGACCCGTGTAAAGGTAGAACGAGACAAAATTATCCTCTGACCTCCACATGTGTAAACATGGTACATGCCTCCCCACATCAATAATGATAAATTTGAAAGGCCTTAAAAAACAACTTAGTGGCCCAGTTCTAAAATGATATCAAGCAAATGACCAAACTTTGAAAGTATAAAGAGAGAAATGCGTAAGGTGTCCTGGGCTTTGCACACTGGGGAATCTTTAGGGAATCTATTCTTGGAATTGAGGTCAGAAAGGTAAACACGTCAGACGAGGCCTTCGGAGCTGTGCTTGAGGTGAGGAGATGGATGGTCAGTCAGGAGATAAGCAAACTCGTTGATTTCGGACAGTGGTGAATGAGTG

Magenta = PCR primer targets

- 5’ Forward primer = TGGAGTGACTCAGCCTTTGC
- 5’ Reverse primer = CACTCATTCACCACTGTCCGA

Cyan = guide RNA target

Black = 5’ breakpoint

**3’ region sequence** = 483 bp

TCCCAGACCCATGCTTTTCTGGGATCATTGATGGCTAGTTTTAGGTTTTCAAAACCGGGAAGAGACGAGATGGCAGTCTAACACGGCTACCCTAGTCTAGGGTGTATTACCTATTTGGAAAGGGGTCCATGTTGCAGAATATGCAAGTCATGCCCAGGATGCGCGGAGGAGACTCCGGTATTGGGTAGAGTTGACTCCTGACCTCTGAGGATTCTTGAAACTCACTCCATAGTATTAGAAAGCAAGGGAGGGTGAAGGGGGAGGTTCACAGGGCTTGAATTCAGAGACAAACATGGGTGGGCAGTCAAGGGGCTACTCCAGTATGGGGCTGCCATAGTGTCTATATTATGCGCAGAAGAATGGGTATTGTTATATTCATTGTCTTCACATCTCTCTCTGGCCTGGCTCATACCTGTATCAGCCTTTGAGGCATCTGTCCCCTTAACCTCAGTGACTCTGATACCAGTCCCCAAGAGTGAGTCG

Magenta = PCR primer targets

- 3’ Forward primer = TCCCAGACCCATGCTTTTCT
- 3’ Reverse primer = CGACTCACTCTTGGGGACTG

Cyan = guide RNA target

Gray = 3’ breakpoint

***Knockout***

**Deletion allele sequence** = 891 bp

TGGAGTGACTCAGCCTTTGCCACCTGGAGCTGTACGGCCTTGGGTCTGTCAGGAATCTCTGAACTTCTTCACACAGCTGCCCAGTGGCTGCCTCTCAGTACACTGGGGAGAACAAGATGAATGAACACAGGGAGCGTTGACATTAGCCCAGGGTATCACTTGGCTTATGTTAGCATGTACAAATTTTTAAATAATTATCTTAACAAAAGTGAGAGGAAAAAAATGGGGAAGGGTGTAGGAGAAACTGGATCGTGGTCTCTTGATTTGATGTGTTATAATTAGGGTTGGGGACACGGCTACTTTTTCATGTTTTTATTTTTTAGAACTAAAACGTGTTAACCATTTTATTTTTTGGAGACATTAGTCCTGGTTGTCCTGGAGCATGTTATGTAGACCGGGCTGGCCTAGAACTTGCAAAGATCCTCCTGCCTCTGCTTTCCCAAGTGCTGGGATTATAGGCATGTATCACCATGCCTGCATATTAATATGTTTTCCGAAAAAGAAGTCTTAGGGGCAGGAGAGATGGCTCAACAGGTAAAGGTACTTGCTGTGCAAGCCTGGTGTCTGAGTTTGATTCCCAGGACCCGTGTAAAGGTAGAACGAGACAAAATTATCCTCTGACCTCCACATGTGTAAACATGGTACATGCCTCCCCACATCAATAATGATAAATTTGAAAGGCCTTAAAAAACAACTTAGTGGCCCAGTTCTAAAATGATATCAAGCAAATGACCAGCTGCCATAGTGTCTATATTATGCGCAGAAGAATGGGTATTGTTATATTCATTGTCTTCACATCTCTCTCTGGCCTGGCTCATACCTGTATCAGCCTTTGAGGCATCTGTCCCCTTAACCTCAGTGACTCTGATACCAGTCCCCAAGAGTGAGTCG

Magenta = PCR primer targets

- PCR product generated using 5’ Forward and 3’ Reverse primers
- 5’ Forward primer = TGGAGTGACTCAGCCTTTGC
- 3’ Reverse primer = CGACTCACTCTTGGGGACTG

Black and Gray = 5’-3’ fusion
